# A Simple, Green Method to Fabricate Composite Membranes for Effective Oil-in-Water Emulsion Separation

**DOI:** 10.3390/polym10030323

**Published:** 2018-03-15

**Authors:** Qianqian Yu, Wenbo Zhang, Xinyue Zhao, Guoliang Cao, Feng Liu, Xin Di, Haiyue Yang, Yazhou Wang, Chengyu Wang

**Affiliations:** Key Laboratory of Bio-Based Material Science and Technology of Ministry of Education, Northeast Forestry University, Harbin 150040, China; yucl2012@126.com (Q.Y.); 474007776@163.com (W.Z.); 15144254883@163.com (X.Z.); mrcaoguoliang@163.com (G.C.); liufeng0517@163.com (F.L.); m13230280636@163.com (X.D.); yanghaiyue2012@163.com (H.Y.); wangyzh53@mail2.sysu.edu.cn (Y.W.)

**Keywords:** corn straw, superhydrophilicity, underwater superoleophobicity, nylon 6,6, phase inversion, oil-in-water emulsion separation

## Abstract

Most factories discharge untreated wastewater to reduce costs, causing serious environmental problems. Low-cost, biological, environmentally friendly and highly effective materials for the separation of emulsified oil/water mixtures are thus in great demand. In this study, a simple, green method was developed for separating oil-in-water emulsions. A corn straw powder (CSP)-nylon 6,6 membrane (CSPNM) was fabricated by a phase inversion process without any further chemical modification. The CSPNM showed superhydrophilic and underwater superoleophobic properties and could be used for the separation of oil-in-water emulsion with high separation efficiency and flux. The CSPNM maintained excellent separation ability after 20 cycles of separation with an oil rejection >99.60%, and the oil rejection and flux have no obvious change with an increasing number of cycles, suggesting a good antifouling property and the structural stability of CSPNM. In addition, the CSPNM exhibited excellent thermal and chemical stability under harsh conditions of high temperature and varying pH.

## 1. Introduction

Nowadays, an increasing number of industries discharge oily wastewater without any treatment, threatening human health and the aquatic ecosystem [1,2]. Emulsified oil/water mixtures generated from most industrial processes, such as petrochemistry, steel production, metal finishing, textile production, food production, and leather production, have a high percentage of oily wastewater [3]. Traditional technologies such as flotation, coalescers, depth filters, centrifugation, and oil-absorbing materials are efficient technologies for separating oil/water mixtures [4,5,6,7]; however, these technologies are ineffective for emulsified oil/water mixtures and surfactant-stabilized emulsions, particularly for emulsions with a droplet size of 20 μm [8]. Although electric field and adding chemicals can demulsify the emulsions, these methods have some disadvantages, such as higher energy consumption and secondary pollution [9]. Therefore, effective techniques to separate oil/water emulsions in wastewater are in great demand. The candidates with the most potential are membrane techniques with advantages such as recycled oil and purified water [10,11,12]. However, the biggest limitation of these materials is easy fouling, caused by the pore plugging, directly leading to a quick decline in flux [13]. 

Recently, membranes and films with special wettability have been used to separate oil/water emulsions and prevent membrane fouling [14,15,16]. It is usually thought that super-wettability can significantly improve the contamination resistance of membranes. Therefore, superhydrophobic separation membranes including polypropylene microfiltration membranes superhydrophilized by co-deposition of dopamine and low-molecular-weight polyethyleneimine [17,18], nano MgO/polyphenylsulfone ultrafiltration composite membranes, and networks gated with a polydimethylsiloxane coating have been reported to separate water-in-oil emulsions [19,20]. Superhydrophilic and underwater superoleophobic separation membranes, such as polypropylene microfiltration membranes decorated with silica, polyelectrolyte/polyvinylidene fluoride (PVDF)-blend membranes, and PVDF membranes decorated with multiwall carbon nanotubes are capable of selectively removing water from emulsified oil/water mixtures [21,22,23,24]. However, the complicated procedures for fabricating polymers, toxicity of reagents, and high cost of materials and equipment limit their practical applications. Therefore, developing low-cost, eco-friendly, and highly effective membrane materials that can separate emulsified oil/water mixtures is of great importance. Recently, many waste and sustainable materials were used as raw materials to fabricate oil/water separation materials to ease environmental stress. For example, Chen et al. used waste fly ash and natural bauxite with the addition of WO_3_ to fabricate highly porous whisker-structured mullite ceramic membranes for separating oil-in-water emulsions [25]; waste potato residue as a feedstock was used to fabricate a mesh for selective oil/water separation, and superhydrophobic corn straw fiber was applied to separate oil/water mixtures [26,27]. Corn straw is also a type of abundant agricultural residue that may cause serious environmental problems if improperly disposed of [28,29]. Thus, using corn straw as a feedstock to fabricate filter membranes may effectively reduce the pollution caused by direct combustion and water, and bring considerable economic benefits.

Herein, we report a green, low-cost, and simple method to fabricate a superhydrophilic and underwater superoleophobic corn straw powder-nylon 6,6 membrane (CSPNM) for emulsion separation. The CSPNM was obtained via a simple phase inversion and could effectively separate micrometer surfactant-free and surfactant-stabilized oil-in-water emulsions. In addition, the CSPNM showed excellent mechanical strength, antifouling, environmental stability, and recyclability, which are essential for practical utilization. 

## 2. Materials and Methods

### 2.1. Materials and Chemicals

Hexane, toluene, anhydrous ethanol and 1,2-dichloroethane (DCE) were purchased from Guangdong Guanghua Sci-Tech Co., Ltd. (Guangdong, China). Sodium hydroxide, hydrochloric acid, and formic acid were provided by Tianjin Kaitong Chemical Reagent Co. (Tianjin, China). All the reagents were of analytical grade and used without any further purification. Diesel was purchased from a local gas station (Harbin, China). Nylon powder (density = 1.06 kg/m^3^) was purchased from Zhenwei Composite Material Limited Company (Shanghai, China). Corn straw was collected from Heilongjiang province, China, crushed, and passed through 400-mesh sieves, followed by washing the corn straw powder with ethanol three times and drying in an oven at 60 °C until constant mass.

### 2.2. Fabrication of Superhydrophilic and Underwater Superoleophobic CSPNMs and Pristine Nylon 6,6 Membranes

Pristine nylon 6,6 membranes (PNMs) and CSPNMs were prepared via the phase-inversion process [30,31,32]. Nylon 6,6 powder (7.47 g) was dissolved in formic acid (50 g) under 3000 rpm stirring to form the nylon 6,6 formic acid solution (NFS). Next, the corn straw (7.47 g) was mixed with the nylon solution under 3000 rpm stirring for 3 h to form CSP-nylon 6,6-concentrated formic acid solution (CSPNFS). Then, the solution was degassed under 40 kPa to remove the gas from the solutions. Approximately 3 min later, the vacuum was released. This process was repeated three times to ensure complete removal of gases. Then, the solution was sealed and kept static for 1 h to get rid of air bubbles. Then, the as-prepared mixture (1.5 mL) was cast onto a pre-cleaned glass pane (12 cm^2^), and the glass pane was immersed into a coagulating bath (500 mL deionized water) to undergo a phase inversion process. NFS and CSNFS were used to fabricate PNMs and CSPNMs, respectively. The resulting membranes were peeled off from the glass pane and rinsed three times with deionized water.

### 2.3. Preparation of the Oil-in-Water Emulsion

Deionized water and oil (*n*-hexane, toluene, or diesel, respectively) were mixed to prepare the surfactant-free oil-in-water emulsions. To obtain the homogeneous emulsions, the mixtures were sonicated at a power of 450 W for 1.5 h. No precipitation was observed in the emulsions under ambient conditions for 24 h. For *n*-hexane-in-water (H/W) emulsion, 4 mL of *n*-hexane were added into 120 mL of water, and the mixture was sonicated. The emulsion droplet size of H/W was in the range of 5–10 μm. For toluene-in-water (T/W) emulsion, 2 mL of toluene were added into 120 mL of water, followed by sonicating the mixture. The emulsion droplet size of T/W is in the range of 3–10 μm. For diesel-in-water (D/W) emulsion, a mixture of 4 mL diesel and 120 mL water was sonicated, resulting in an emulsion droplet size in the range of 5–10 μm. The emulsion droplet sizes of all the emulsions were determined by optical microscopy and found to be in the range of 2–10 μm. Surfactant-stabilized oil-in-water emulsions were prepared by adding surfactant and oil into deionized water under stirring. In detail, tween 80-stabilized *n*-hexane-in-water emulsion (T/H/W was prepared by mixing *n*-hexane and water (1:99, *v*/*v*) with adding 0.1 mg of tween 80 per mL of emulsion and stirring under 3000 rpm. A tween 80-stabilized toluene-in-water (T/T/W) emulsion was prepared by mixing toluene and water (1:99, *v*/*v*) adding 0.1 mg tween 80 per mL of emulsion and stirring at 3000 rpm. A tween 80-stabilized diesel-in-water (T/D/W) emulsion was prepared by mixing diesel and water (1: 99, *v*/*v*) with adding 0.1 mg of tween 80 per mL of emulsion and stirring under 3000 rpm [33]. All the emulsions could be stable for more than one month without demulsification or precipitation. The emulsion droplet sizes ranged from several hundred nanometers to 5 μm.

### 2.4. Emulsion Separation Experiment

The as-prepared CSPNM was sealed within the filter apparatus. The 10 mL of feed emulsion were poured onto the membrane and then the filtrate was collected with a driving pressure of 0.01 MPa (vacuum −0.1 bar). The flux was determined by calculating the following formula [34,35,36]:

(1) $$\mathrm{Flux}=\frac{V}{At}$$

where *V* is the volume of the solution passing through the membrane, *A* is the valid area of the membrane (1.77 cm^2^), and *t* is the time of the solution passing through the membrane. Original emulsions and their corresponding collected filtrates after a one-time separation were measured by UV–Vis spectroscopy and total organic carbon (TOC) analyzer. The oil content in water was measured using a TOC analyzer, and the characteristic peak of the oil was measured using a UV–Vis spectroscopy. The average value of five samples was measured. In order to study the separation ability of the CSPNM deeply, the oil rejection was determined by calculating the ratio of the rejected oil content in the feed solution by the membrane to the oil content in the feed solution using the follow formula [37]:

(2) $$R=\left(\frac{C_{f1}-C_{f2}}{C_{f1}}\right)\times 100$$

where *R* is the oil rejection of the CSPNM, *C*_f1_ is the oil content in feed solution, and *C*_f2_ is the oil content in filtration.

### 2.5. Continuous Separation

The as-prepared CSPNM was sealed between a vertical glass tube and a conical flask, and then the CSPNM was pre-wetted with water. Water (10 mL) and the H/W emulsion (10 mL) were poured into two different filter apparatuses and the filtration was driven by a vacuum pump at a pressure of 0.01 MPa (vacuum −0.1 bar). The whole feed solution permeated the membrane, and a second cycle followed. During each cycle 10 mL feed solution were added. The time it took for the solution to pass through the membrane was recorded by a timer. The flux was calculated by Formula (1) (where *V* is the volume of feed solution passing through the membrane, *t* is the time consumed by a solution of a certain volume passing through the membrane, and *A* is the valid area of the membrane (1.77 cm^2^)). The filtrate was measured by total organic carbon (TOC) analyzer and then the oil rejection was calculated by Formula (2), where *C*_f1_ is the oil content in the feed solution and *C*_f2_ is the oil content in filtration.

### 2.6. Cycle Experiment

Feed solution (10 mL) was poured into the apparatus and the filtrate was measured by total organic carbon (TOC) analyzer, while the time of solution passing through the membrane was recorded by a timer. After one cycle, the membrane was washed with ethanol (10 mL), cleaned, and dried at 70 °C (the test used the same sample to directly reflect the variation trend). The above process was repeated 20 times. 

### 2.7. Environmental Durability Experiments 

The CSPNM was heated at different temperatures for 12 h to test the thermal stability of the CSPNM. The oil contact angles (CAs) and roll-off angles of the CSPNM were measured underwater after the CSPNM stayed at 0 °C to 120 °C for 12 h. The CSPNMs were in contact with corrosive aqueous solutions (HCl and NaOH aqueous solutions, pH 1 to 14) for 30 h to study the chemical durability. Then, the oil CAs and roll-off angles of the membranes were measured underwater.

### 2.8. Characterizations

The morphology of the as-prepared CSPNM was investigated by scanning electron microscopy (SEM, TM3030, Tokyo, Japan). The water CAs were measured using 5 μL of deionized water droplets, and the oil CAs underwater were measured using 5 μL of DCE droplets after immersing the CSPNM into water at room temperature, which was conducted on an OCA20 instrument (Data-physics, Stuttgart, Germany). The tensile strength of the membranes was measured using a tensile testing machine (AI-7000S, Gotech testing machines, Dongwan, China). Feed solutions, filtrates, and emulsifier aqueous solutions were analyzed by optical microscopy (Eclipse 80i, Tokyo, Japan), a UV–Vis spectrometer (Cary 100, Agilent, Melbourne, Australia), and total organic carbon analysis (TOC-VCPN, Shimadzu, Kyoto, Japan), respectively.

## 3. Results

### 3.1. Membrane Characterization

**Figure 1:**
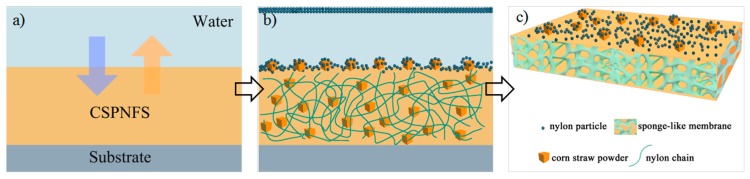
Formation of the CSPNM (corn straw powder-nylon 6,6 membrane) by a phase-inversion process.

The CSPNM was fabricated via the phase inversion using water as a coagulation bath. Figure 1a–c show the schematic fabrication process of the membrane. As shown in Figure 1a, the CSPNFS was cast onto the glass pane and immersed into deionized water, where the solvents were immediately exchanged between the water and formic acid. During the phase-inversion process, the water will take some of the insecure nylon particles (INP) away from the surface of the CSPNM, as shown in the top of Figure 1b. The addition of CSP diminished the INPs, forming a sponge-like membrane structure (Figure 1c) [38].

**Figure 2:**
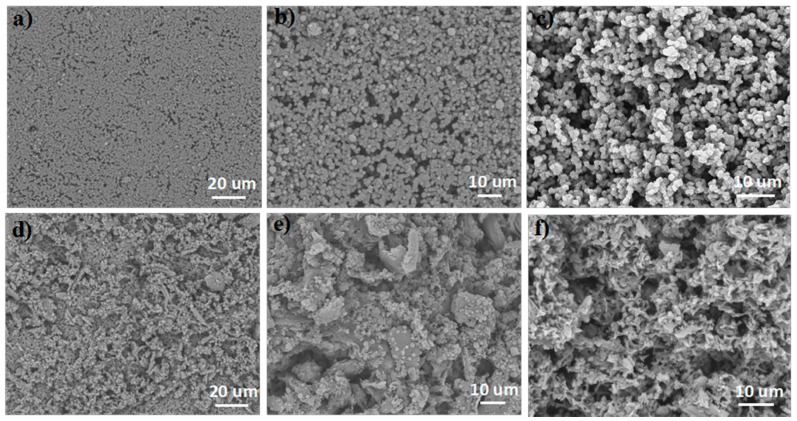
SEM images of the top surface (**a**,**b**); cross sections of the pure nylon membrane (**c**) and the top surface (**d**,**e**); and cross section of the CSPNM (**f**).

Figure 2 shows the morphology and microstructure of the PNM and the CSPNM. The details of cross section, top surface, and bottom surface of the CSPNM are shown in Figure S1 of the Supplementary Materials. Figure 2a shows many micro-flaws on the top surface of the PNM, and obvious flaws were observed, as shown in Figure 2b. The cross section of the PNM (Figure 2c) shows many particles piled together. 

**Figure 3:**
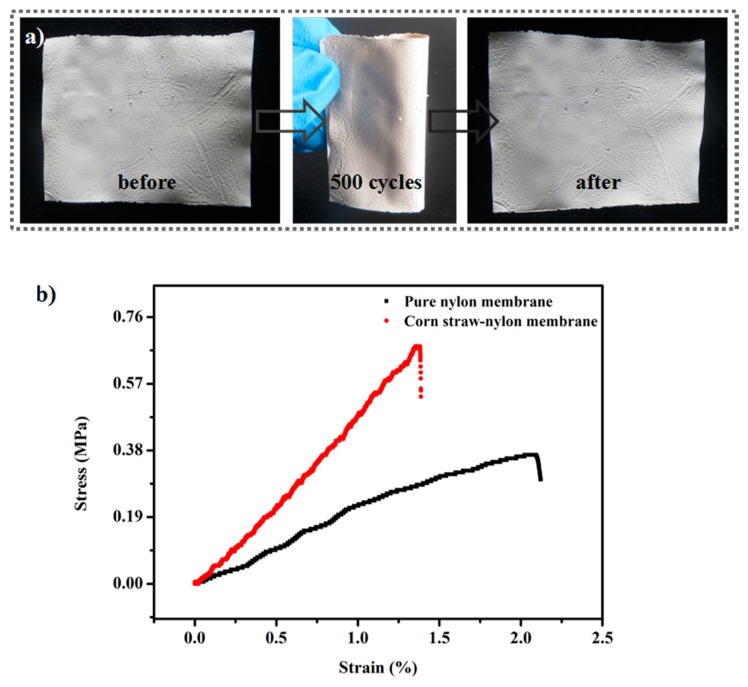
(**a**) Optical images of CSPNM before and after being bent over 500 times; (**b**) stress–strain curves of the PNM (pristine nylon 6,6 membrane) and the CSPNM, respectively.

The addition of the CSP improved the morphology and microstructure of the membrane. We consider that the CSP plays two distinct roles in the CSPNM. First, CSP reduces the number of INPs to prevent nylon particles (NPs) from leaving, so more nylon will participate in the formation of the structure. Secondly, Figure 3d shows a rougher structure at the top surface of the CSPNM. According to the Wenzel model [39], the hydrophilicity of a hydrophilic solid substrate will be enhanced by surface roughness because of the capillary effect. The high-magnification image (Figure 2e) clearly shows the microstructure. Moreover, the microstructure changed from a particles-accumulation structure to a continuously spongy structure, as shown in Figure 2f. During the phase-inversion process, the morphology of nylon transformed with the addition of CSP, thereby compensating for the flaws.

To test the flexibility of CSPNM, the membrane was rolled up and released over 500 times. As shown in Figure 3a, there were no cracks after the bending cycles, indicating the superior mechanical performance and flexibility of the CSPNM. The PNM and the CSPNM were cut into 30 × 10 mm^2^ pieces to determine the tensile strength, as shown in Figure 3b. The PNM has a tensile strength of 0.377 MPa and an elongation of 2.2%, respectively. The CSPNM showed higher mechanical strength with a tensile strength of 0.689 MPa and lower elongation of 1.4%, attributed to the structural transformation from the particles-accumulation structure to the sponge-like structure [40]. The binding strength between nylon particles (NPs) in the PNM is weaker, thus leading to inferior mechanical performance. The stronger binding strength between CSP and nylon improves the mechanical performance of the CSPNM, suggesting that the sponge-like structure of the CSPNM is more stable than the particles-accumulation structure of the PNM. The lower elongation of the CSPNM showed that the CSPNM has better dimensional stability, suitable for repeated use. These results indicate the excellent mechanical performance and flexibility of the CSPNM.

**Figure 4:**
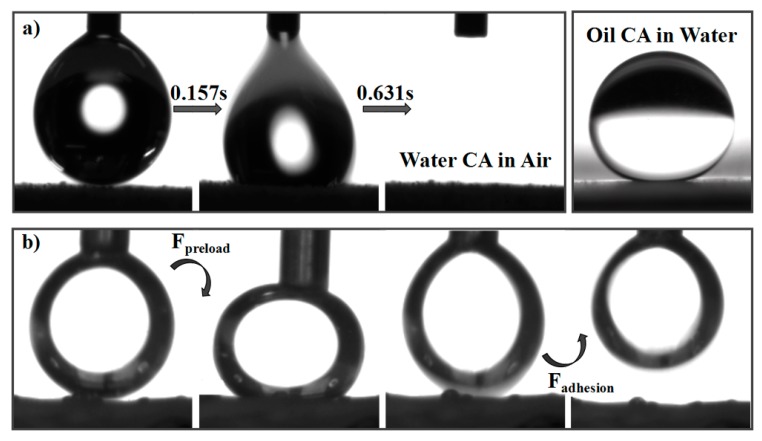
(**a**) Wettability of the CSPNM toward (**left**) water in air and (**right**) oil under water; (**b**) photographs of underwater oil contact the CSPNM.

Figure 4a shows the wetting behavior of water and oil on the top surface of the as-prepared CSPNM. A water droplet (5 μL) spreads out and permeates into the CSPNM within 0.78 s when it comes into contact with the membrane (Movie S1), and a water CA of nearly 0° in the air is observed (Figure 4a, left), indicating the high hydrophilicity of the CSPNM. The oil droplet attained a quasi-sphere on the underwater surface of the membrane with a CA of 157°, showing the underwater superoleophobicity of the membrane (Figure 4a, right). When the underwater CSPNM comes into contact with oil, the water trapped in the rough structures will significantly decrease the contact area between the surface of the membrane and oil droplet, showing a large oil CA underwater (underwater superoleophobicity). Thus, the CSPNM shows superhydrophilic and underwater superoleophobic properties.

To better assess the membrane’s oil repellency underwater, its adhesion behavior was measured during the process of oil contact with the membrane surface underwater. Before the beginning of the experimentation, an oil droplet (5 μL) was squeezed on the CSPNM surface underwater under a preload, and then the oil droplet was released. In the relaxing process, the oil droplet maintains the spherical shape underwater, as shown in Figure 4b, indicating that CSPNM displays an ultralow adhesion with the oil droplet underwater. The results demonstrate that the CSPNM has a much better anti-adhesion performance to oil underwater, probably attributed to the formation of hydrogen bonds between the water and hydrophilic components in the CSPNM [35].

### 3.2. Filtration

**Figure 5:**
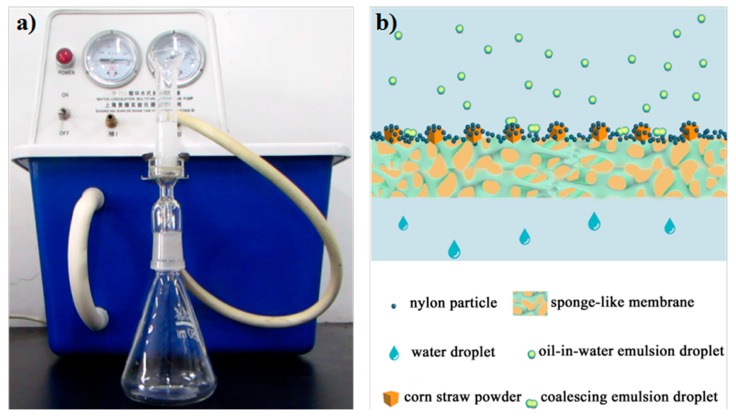
Schematic of the demulsification process of the emulsion on the CSPNM.

The filtration system is shown in Figure 5a. The as-prepared CSPNM was sealed between a vertical glass tube and a conical flask. After pre-wetting the CSPNM with water, the oil-in-water emulsion was poured into the glass tube and the emulsion was separated at a low pressure (0.1 bar) by a vacuum pump. The separation process is shown in Movie S2. A schematic is shown in Figure 5b for a clear understanding of this separation process. The emulsion comes into contact with the top surface of the CSPNM and will demulsify once the emulsion droplets touch the membrane surface. The water phase passes through the CSPNM and meanwhile the oil droplets block and coalesce at the surface of the membrane, as shown in Figure 5b. The wetting behavior of the membrane transformed from superhydrophilicity in the air to superoleophobicity underwater after being prewetted by water. The water layer formed at the interface of oil and the membrane, which repelled the oil.

**Figure 6:**
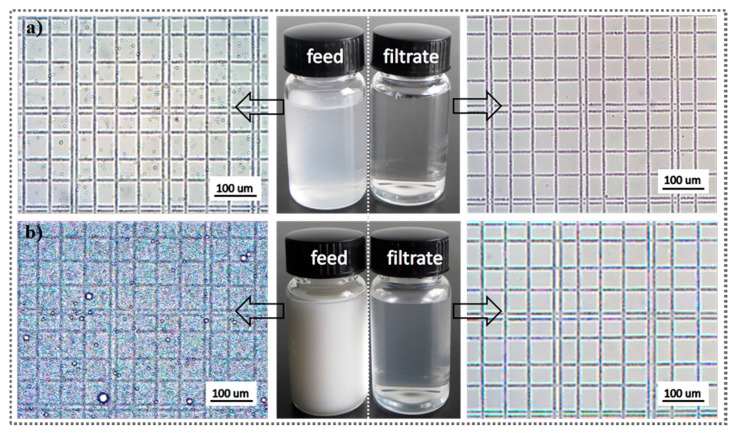
Separation results for various oil-in-water emulsions: (**a**) surfactant-free H/W (*n*-hexane-in-water) emulsion and (**b**) D/W (diesel-in-water) emulsion.

To better understand the separation capability of the CSPNM, a series of oil-in-water emulsions including surfactant-free/surfactant-stabilized emulsions were used for this separation. Optical microscopy was used for observing different feed solutions and their corresponding permeate filtrates. Figure 6a shows the separation results of the H/W emulsion as an example. In the middle photograph, the transparent collected filtrate (right) contrasts with the original white feed solution (left). The emulsion droplets of micrometer size were observed in the feed solution, and no emulsion droplets were observed in the image of the collected filtrate. For the D/W emulsion, the entire view shows densely packed droplets (Figure 6b), whereas no droplets were observed in the photo of the collected filtrate, indicating the successful removal of diesel. The separate results of other emulsions are shown in Figure S2, and none of the images of the filtrates show any droplet, implying the good separation capability of the CSPNM for various emulsions. 

**Figure 7:**
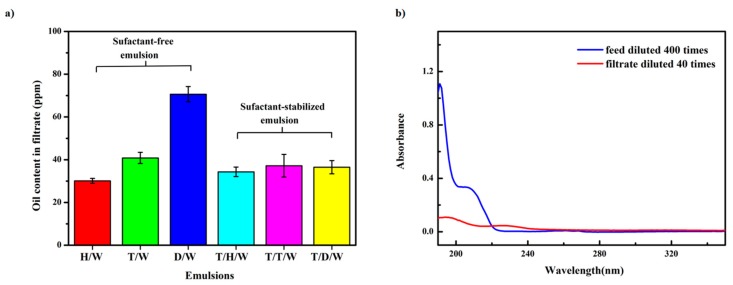
Oil-in water emulsion separation results of the CSPNM. (**a**) Oil content in the filtrates for a series of emulsions; (**b**) UV–Vis spectra for the T/W (toluene-in-water) emulsion diluted 400 times and the filtrate diluted 40 times.

To further study the separation ability of the CSPNM, the collected filtrates were analyzed by UV–Vis spectroscopy and TOC analysis. For each surfactant-stabilized emulsion, the oil plus the surfactant residues in the filtrate is the TOC value. As shown in Figure 7a, the oil contents of the other emulsions were <45 ppm except for the D/W emulsion, indicating the high separation efficiency of the CSPNM. The D/W emulsion showed higher oil content, probably because of the densely packed emulsion droplets flooding the feed solution (Figure 6b, left). 

The oil rejection of the CSPNM directly reflects the separation effect of the membrane. The oil contents of the original emulsions and the oil rejection of the CSPNM for various oils are listed in Table S1. The oil concentration in the original emulsions ranges from 12,200 ppm to 33,360 ppm, and their corresponding oil rejections are >99.60%; some of them are even up to 99.85%, illustrating the high separation efficiency of the CSPNM. 

UV–Vis spectrometry was used to analyze the emulsions and their corresponding collected filtrates. As shown in Figure 7b, no obvious characteristic peaks for toluene are observed in the spectrum of the filtrate. Similar results are also obtained for the H/W and D/W emulsions (Figure S3).

**Figure 8:**
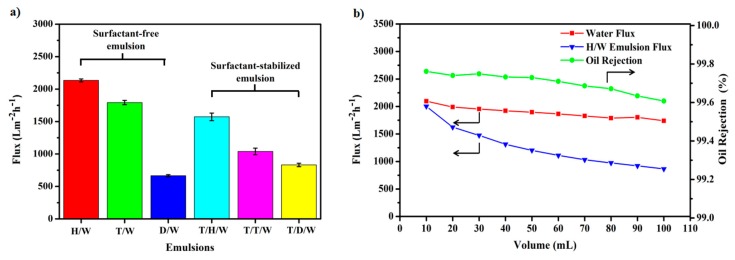
(**a**) Permeate flux for various emulsions of the CSPNM; (**b**) continuous separation flux for water and H/W emulsion of the CSPNM, and oil rejection of the CSPNM for continuous separating H/W emulsion.

As shown in Figure 8a, emulsions exhibit high fluxes with 0.1 bar of applied pressure across the CSPNM, the fluxes of surfactant-free H/W, T/W and D/W emulsions are 2133.33 ± 22.34, 1792.64 ± 32.58 and 666.39 ± 16.78 L·m^−2^·h^−1^, respectively. The fluxes of tween 80-stabilized T/H/W, T/T/W, and T/D/W emulsions are 1572.54 ± 58.18, 1039.28 ± 50.61, and 831.73 ± 26.62 L·m^−2^·h^−1^, respectively. Additionally, the lower flux of D/W emulsion was determined by the high distribution of oil droplets in the emulsion (Figure 5b, left), decreasing the flux of the D/W emulsion. Figure 8b shows the results of the continuous separation. The decrease of the water flux was not obvious, and the flux decreased by 17.4% from the 10 mL volume to 100 mL. For the H/W emulsion, the flux decreased from 10 mL of volume (about 2002.65 L·m^−2^·h^−1^) to 20 mL of volume (about 1624.15 L·m^−2^·h^−1^), and the flux decreased by 9.2% from the 20 mL volume to 30 mL. Each time an increase of feed solution (10 mL), the flux decrease ratio was <10%. The oil rejections of the CSPNM for continuous separating H/W emulsion were >99.60%; the oil rejection decreased from 10 mL of volume (about 99.76%) to 100 mL of volume (about 99.60%), indicating a good separation efficiency of the CSPNM.

### 3.3. Recyclability and Chemical Durability Test Experiments

**Figure 9:**
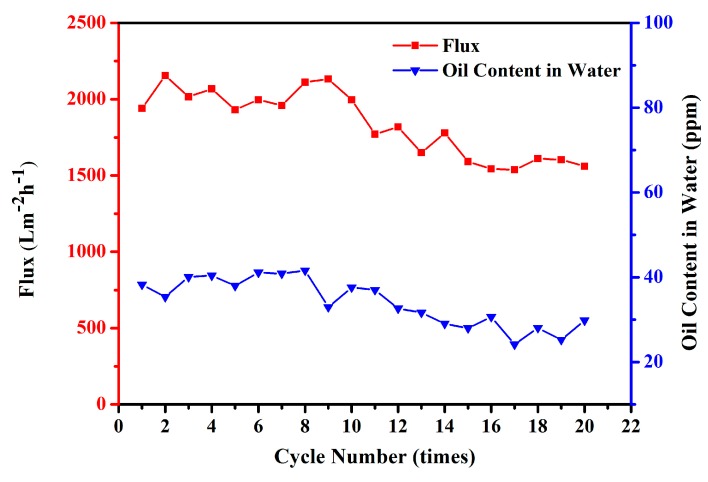
Cycling performance of the CSPNM using *n*-hexane-in-water emulsion as model feed liquid.

To test the recyclability and the antifouling property of the CSPNM, a cyclic experiment was conducted, using 10 mL of feed emulsion (H/W) in every cycle. Figure 9 shows the flux and oil content in the filtrates within 20 cycles. In the whole testing process, the flux decreased by 19.54% from the first cycle (about 1940.17 L·m^−2^·h^−1^) to the 20th cycle (about 1561.09 L·m^−2^·h^−1^); no obvious flux decrease or oil content decrease were observed at the near cycle, indicating the superior recyclability and good antifouling property of the membrane.

**Figure 10:**
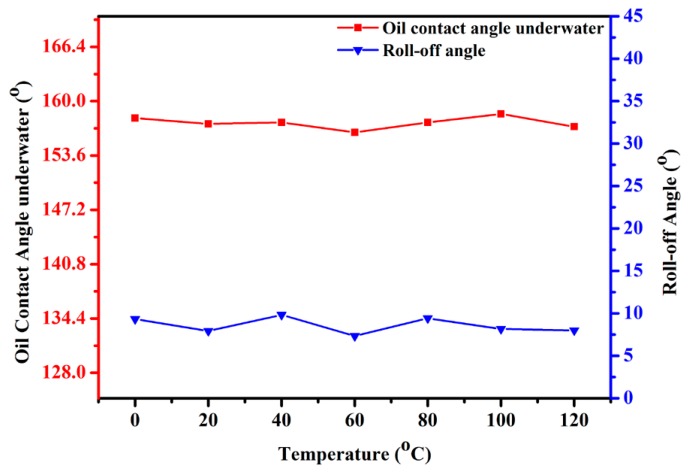
Variation in the underwater oil CA and roll-off angles of the CSPNM with temperature increase.

To study the thermal stability of the CSPNM, the underwater oil CAs and roll-off angles of the CSPNM were measured after heating at different temperatures for 12 h. Figure 10 shows that the CSPNM maintains its superwetting property after the heating processes with the oil CAs >150° and roll-off angles <15°. These results indicate the superior thermal stability of the CSPNM. 

**Figure 11:**
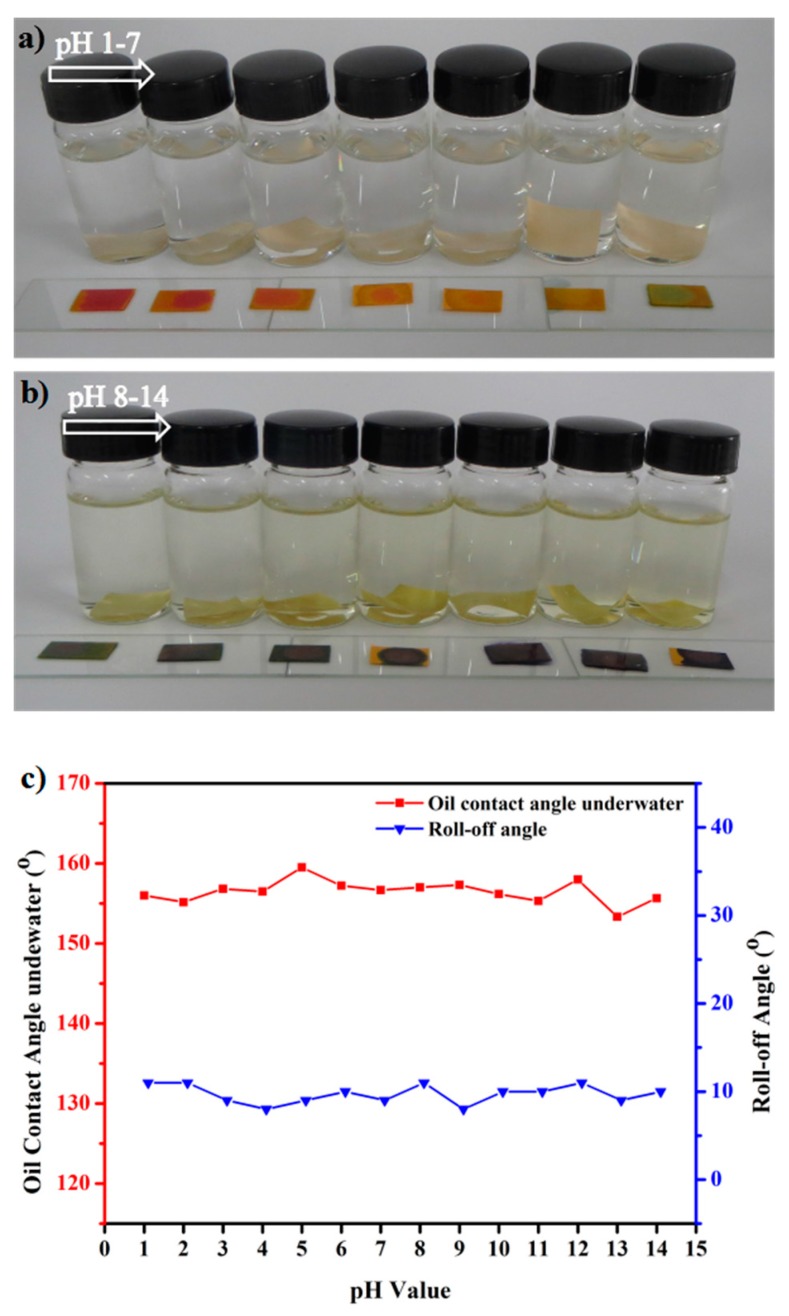
The CSPNM after being immersed in solutions with different pH values: (**a**) pH 1 to 7; and (**b**) pH 8 to 14. (**c**) Variation in the oil CAs under water and water CAs of the membrane with increasing pH.

The chemical durability of the CSPNM was tested by measuring the underwater oil CAs and roll-off angles of the membrane after immersing CSPNMs in solutions with different pH values (Figure 11a,b) for 30 h. Figure 11c shows the CSPNM with an underwater oil CA >150° and roll-off angles <15° after being in contact with the corrosive aqueous solutions (1 to 14 pH values), showing the superior environmental stability of the CSPNMs under harsh conditions.

## 4. Conclusions

In summary, superhydrophilic and underwater superolephobic CSPNMs were fabricated by a simple and environmentally friendly phase-inversion method. The addition of CSP significantly improved the structure and mechanical strength of the membrane. In addition, oil-in-water emulsions were separated with high oil rejection (>99.60%) and flux >660.00 L·m^−2^·h^−1^. After 20 separation cycles of the H/W emulsion, the oil rejection was still >99.50% and no obvious decline in the flux was observed, indicating superior recyclability. Moreover, the CSPNM maintained underwater superolephobicity after being immersed in various corrosive aqueous solutions and exposed to different temperatures, exhibiting excellent environmental stability. We believe that the utilization of CSP and emulsion separation would be of great significance to sustainable development.

## Supplementary Materials

The following information is available online at http://www.mdpi.com/2073-4360/10/3/323/s1, Table S1: the oil content in the feed solutions and corresponding oil rejection, Figure S1: the details of the cross section, top surface, and bottom surface of the CSPNM, Figures S2 and S3: the separation results for various oil-in-water emulsions. Supplementary video: a water droplet permeates into the CSPNM (Video S1) and the separation process (Video S2).
