# Supplementary material for: A Simple, Green Method to Fabricate Composite Membranes for Effective Oil-in-Water Emulsion Separation
Source: Polymers (Basel). 2018 Mar 15;10(3):323. doi: 10.3390/polym10030323 (PMC6415185; doi:10.3390/polym10030323)
Supplement: Supplementary file 1 [file polymers-10-00323-s001.zip › polymers-259948-supplementary/polymers-259948-supplementary.pdf]

Supporting Information (SI)

A Simple, Green Method to Fabricate Composite Membranes for Effective Oil-in-Water Emulsion Separation

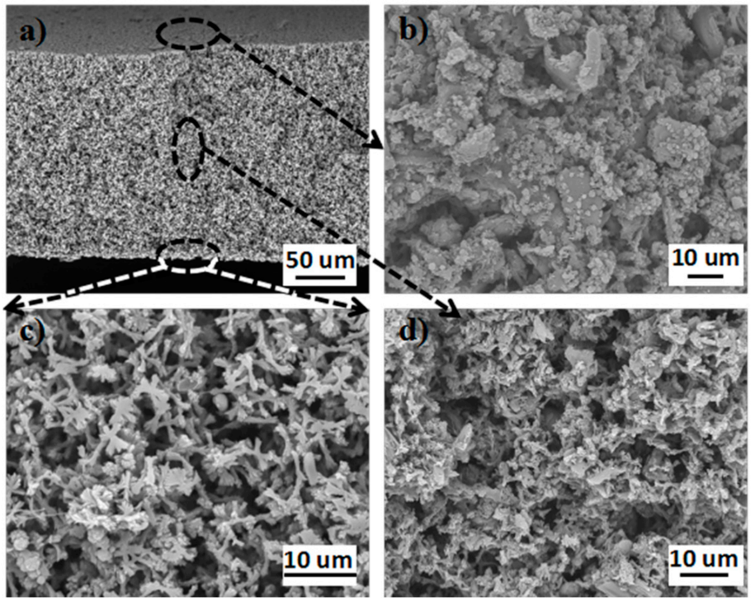

Figure S1. SEM images of cross section (**a**,**d**), top surface (**b**), and bottom surface (**c**) of the CSPNM.

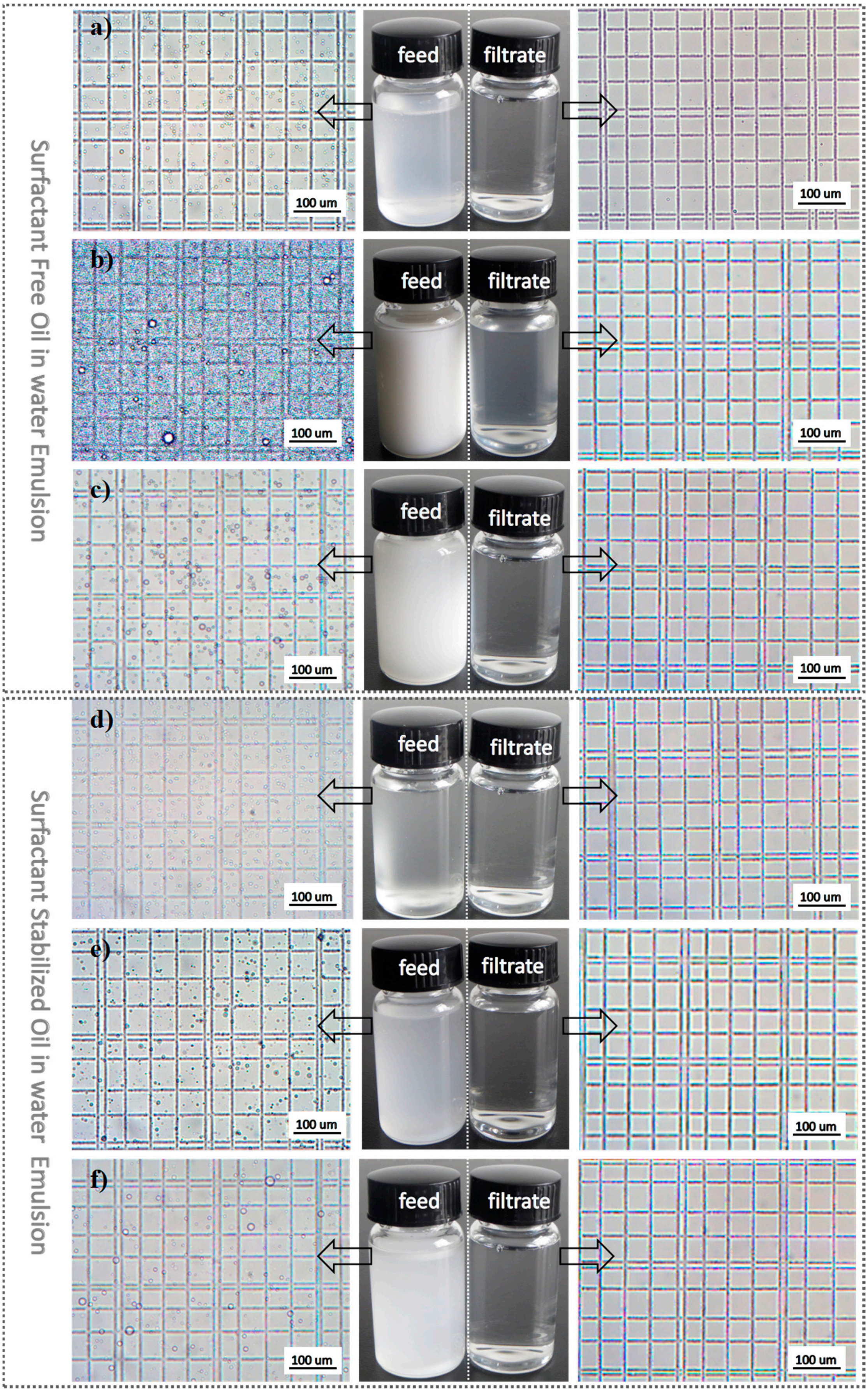

Figure S2. Separation results for various surfactant-free oil-in-water emulsions:

n-hexane-in-water (H/W) (**a**), toluene-in water (T/W) (**c**), and diesel-in-water emulsion (D/W) (**e**). Separation results for various surfactant-stabilized oil-in-water emulsions: n-hexane-in-water (S/H/W) (**b**), toluene-in water (S/T/W) (**d**), and diesel-in-water emulsion (S/D/W) (**f**). All the filtrates become transparent and no droplet is observed in the corresponding optical image.

Table S1. Oil rejection of the CSPNM and content in the feed solutions.

Emulsion	Content of oil in feed solutions (mg/L)	Rejection of the CSPNM (%)
n-hexane -in- water (H/W)	12200	99.75
n-hexane -in- water (T/H/W)	23600	99.85
toluene -in- water (T/W)	12840	99.68
toluene -in- water (T/T/W)	23650	99.84
diesel -in- water (D/W)	33360	99.79
diesel -in- water (T/D/W)	24730	99.84

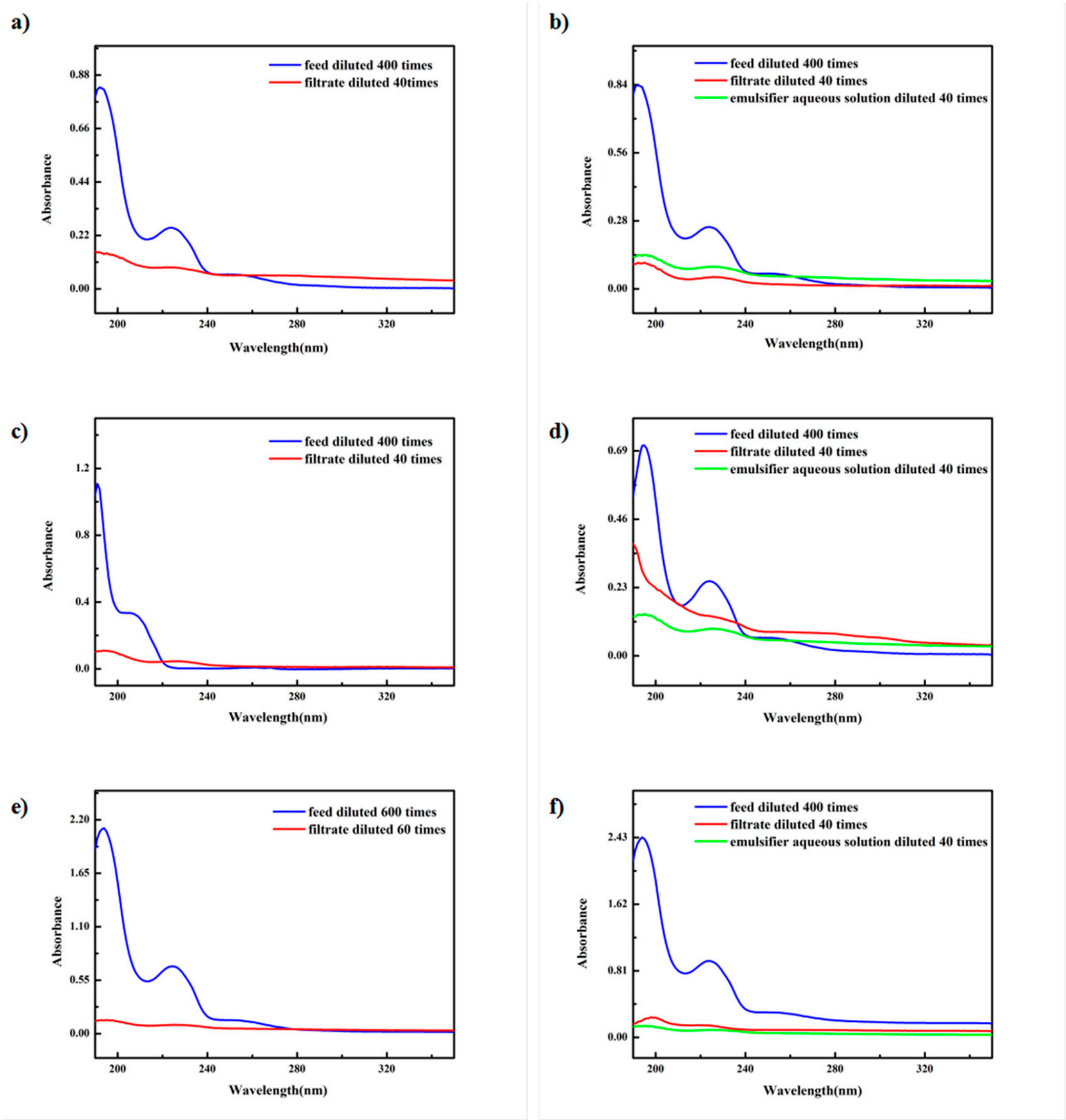

Figure S3. UV-VIS spectra for n-hexane-in-water emulsion (H/W) (**a**), n-hexane-in-water emulsion (T/H/W) (**b**), toluene-in-water emulsion (T/W) (**c**), toluene-in-water emulsion (T/T/W) (**d**), diesel-in-water emulsion (D/W) (**e**), and diesel-in-water emulsion (T/D/W) (**f**).
